# 3,4-Dihydroxiphenylacetic Acid-Based Universal Coating Technique for Magnetic Nanoparticles Stabilization for Biomedical Applications

**DOI:** 10.3390/jfb14090461

**Published:** 2023-09-06

**Authors:** Alevtina Semkina, Aleksey Nikitin, Anna Ivanova, Nelly Chmelyuk, Natalia Sviridenkova, Polina Lazareva, Maxim Abakumov

**Affiliations:** 1 Department of Medical Nanobiotechnology, N.I. Pirogov Russian National Research Medical University, 117997 Moscow, Russia; alevtina.semkina@gmail.com (A.S.); nikitin.aa93@mail.ru (A.N.); super.fosforit@yandex.ru (A.I.); nellichmelyuk@yandex.ru (N.C.); polizara604@gmail.com (P.L.); 2 Department of Basic and Applied Neurobiology, Serbsky National Medical Research Center for Psychiatry and Narcology, 119991 Moscow, Russia; 3 Laboratory of Biomedical Nanomaterials, National University of Science and Technology (MISIS), 119049 Moscow, Russia; 4 Department of General and Inorganic Chemistry, Mendeleev University of Chemical Technology of Russia, 125047 Moscow, Russia; laimik231@yandex.ru

**Keywords:** magnetic nanoparticles, 3,4-dihydroxiphenylacetic acid, nanotechnology, functional coatings, stabilization, surface modification, colloidal stability, phase transfer

## Abstract

Magnetic nanoparticles based on iron oxide attract researchers’ attention due to a wide range of possible applications in biomedicine. As synthesized, most of the magnetic nanoparticles do not form the stable colloidal solutions that are required for the evaluation of their interactions with cells or their efficacy on animal models. For further application in biomedicine, magnetic nanoparticles must be further modified with biocompatible coating. Both the size and shape of magnetic nanoparticles and the chemical composition of the coating have an effect on magnetic nanoparticles’ interactions with living objects. Thus, a universal method for magnetic nanoparticles’ stabilization in water solutions is needed, regardless of how magnetic nanoparticles were initially synthesized. In this paper, we propose the versatile and highly reproducible ligand exchange technique of coating with 3,4-dihydroxiphenylacetic acid (DOPAC), based on the formation of Fe-O bonds with hydroxyl groups of DOPAC leading to the hydrophilization of the magnetic nanoparticles’ surfaces following phase transfer from organic solutions to water. The proposed technique allows for obtaining stable water–colloidal solutions of magnetic nanoparticles with sizes from 21 to 307 nm synthesized by thermal decomposition or coprecipitation techniques. Those stabilized by DOPAC nanoparticles were shown to be efficient in the magnetomechanical actuation of DNA duplexes, drug delivery of doxorubicin to cancer cells, and targeted delivery by conjugation with antibodies. Moreover, the diversity of possible biomedical applications of the resulting nanoparticles was presented. This finding is important in terms of nanoparticle design for various biomedical applications and will reduce nanomedicines manufacturing time, along with difficulties related to comparative studies of magnetic nanoparticles with different magnetic core characteristics.

## 1. Introduction

Magnetic nanoparticles (MNPs) capture attention within many different biomedical applications, such as magnetic resonance imaging (MRI), drug delivery, magnetic hyperthermia, magnetofection, biosensing theranostics, etc. [1,2,3]. It is well known that size, shape, and magnetic properties strongly affect the application of nanoparticles in biomedicine [4,5,6,7], such as their MRI contrast properties, magnetic hyperthermia efficiency, and pharmacokinetics; however, surface coating may play a no less important role [8,9,10]. Usually, unmodified nanoparticles tend to aggregate due to a list of reasons, such as gravity, large surface energy, and magnetic interactions [11]. Coating by biocompatible materials with the appropriate chemical nature and increasing electrostatic and steric repulsions results in stable colloid formation [12,13]. The formation of such stable solutions was shown for many different biocompatible coatings, e.g., dextran, polyvinyl alcohol (PVA), silica, dimercaptosuccinic acid (DMSA), and many others [14,15,16,17,18,19,20,21,22,23,24,25].

Magnetic properties of nanoparticles mostly depend on the structure, size, and shape of the magnetic core [26,27,28], whereas the chemical structure of a surface coating mostly dictates its colloidal stability and plays an important role in interaction with cells and tissues in in vitro and in vivo experiments [9,29,30]. Optimization of both parameters is crucial for the increase of MNP effectiveness in biomedical applications. On the other hand, a big diversity of synthetic procedures of magnetic cores, such as coprecipitation, thermal decomposition in organic solvents, sol-gel method, and many others, leads to the synthesis of magnetic cores with different surface properties. For example, MNPs obtained by the coprecipitation method usually possess a hydrophilic surface [31,32], whereas MNPs obtained by the thermal decomposition method are usually coated with hydrophobic molecules and are not soluble in water [33]. Furthermore, high-energy milling, pyrolysis, chemical vapour deposition or electron-beam lithography results in MNPs which are in the form of dry powders [34]. In order to obtain stable water–colloidal solutions of MNPs obtained by each of these techniques, it is necessary to develop a special coating procedure. For example, a dextran coating can be successfully used for the stabilization of hydrophilic MNPs obtained by the coprecipitation technique, but it does not allow to stabilize MNPs synthesized by the thermal decomposition route, because dextran is insoluble in organic solvents. Whereas MNPs obtained by thermal decomposition are coated with hydrophobic surfactants, it does not allow them to be transferred to water solutions. This leads to a situation where, for each type of magnetic core synthesis, a special coating procedure has to be developed. This approach has some obvious disadvantages: (1) for each new synthetic approach being developed, a new type of coating technique must be developed, or one of the existing ones has to be modified; (2) different coatings lead to changes in the interaction of MNPs in in vitro and in vivo experiments, even for MNPs with the same size and shape of their magnetic core; (3) a diversity of MNPs sizes, shapes, compositions, and their combination with a variety of coatings do not allow for performing analysis of the MNPs properties as too many variables have to be taken into account. Therefore, the idea of developing a coating for MNPs that provides (1) biocompatibility; (2) strong bonding with MNP surface; (3) high colloidal stability; (4) functional groups for further modification; and (5) can be used for all types of MNPs despite its synthetic route seems extremely relevant as a well-tuned tool for various biomedical applications.

Catechol derivatives, such as dopamine, L-3,4-dihydroxyphenylalanine (L-DOPA), or DMSA, have become widespread due to their chemical bifunctionality and specific ability to form coordination bonds with metal atoms on the surface of MNPs [35,36,37]. In addition, their mild oxidation leads to polymerization and the subsequent formation of a polymer-like material, and this process can be implemented for the stabilization stage [38]. However, dopamine contains free NH_2_ groups providing a positive surface charge of functionalized MNPs, leading to possible cytotoxic effects and unspecific binding to cells [39]. In this regard, it is advisable to seek another analogue to use as a coating material.

Here, we present a new coating–ligand exchange technique allowing for obtaining water–colloidal solutions of MNP by using 3,4-dihydroxiphenylacetic acid (DOPAC), another catechol derivative, as a perspective stabilization agent. For the first time, we have shown that this technique allows the stabilization of MNPs synthesized by both coprecipitation and thermal decomposition techniques with various shapes, sizes, and compositions which makes the proposed approach universal. Moreover, the stabilization of MNPs by DOPAC does not affect their magnetic properties, allowing them to be further used for MRI, magnetic hyperthermia, drug delivery, and magnetomechanical applications.

## 2. Materials and Methods

### 2.1. Synthesis of CoFe_2_O_4_ and Fe_3_O_4_ NPs by Thermal Decomposition Method

The mixture of reagents (Table 1) was placed in a 250 mL three-necked round bottom flask, equipped with a reflux condenser and thermometer. First, the reaction mixture was heated up to 130 °C under argon flow and kept for 30 min. Then, the mixture was heated up to 280 °C with a rate of 3 °C/min and kept for another 2 or 4 h. After cooling the solution to room temperature, nanoparticles were separated from the solution by centrifugation for 30 min at 6000 rpm, after which the formed precipitate was redissolved in toluene.

### 2.2. Synthesis of CoFe_2_O_4_ NPs by Co-Precipitation Method

In 20 mL of deionized water containing 0.5 mL of HCl, 10 mmol of FeCl_3_ and 5 mmol of CoCl_2_·6H_2_O were dissolved. The resulting solution was added dropwise to 100 mL of 1M NaOH and preheated to the boiling point. The boiling of the solution was maintained for 30 min with continuous vigorous stirring of the reaction mixture. After that, the reaction mixture was cooled to room temperature, and the NPs were isolated through the permanent magnet. The residue was dissolved in deionized water and the pH was adjusted to 7.0–8.0 by repeated washing using centrifugal filters (Millipore Amicon Ultra-4, MWCO 30 kDa). Finally, dry NP powder was prepared using a vacuum evaporator. To stabilize NPs in nonpolar solvents, 5 mg of such powder were dissolved in a mixture of 2 mL of toluene and 2 mL of oleic acid, followed by sonication with an ultrasonic probe (30 W) in pulse mode (1 s pulse/1 s pause) for 2 h. After incubation overnight, NPs were isolated by centrifugation (14,000 rpm, 20 min) and dispersed in 10 mL of toluene.

### 2.3. Fe_3_O_4_-Au Nanoparticles Synthesis

The Fe_3_O_4_-Au NPs were synthesized following a thermal decomposition method and according to a modified protocol [40]. Briefly, 0.9 mL of Fe(CO)_5_ were injected into the pre-heated (120 °C) mixture of octadecene-1 (20 mL), oleic acid (1.9 mL), and oleylamine (2.0 mL) under the argon atmosphere. After 3 min, the solution, which consists of 40 mg of tetrachloroaurate (III) three hydrate, 5 mL octadecene-1 and 0.5 mL oleylamine, was injected. Then the reaction mixture was heated to the boiling point at a rate of 3 °C per minute, kept for 45 min, and cooled to room temperature, followed by 1 h of room-temperature oxidation in ambient air. The nanoparticles were isolated via centrifugation (5030× *g*, 20 min, 3–4 times), washed with isopropanol, and dispersed in hexane.

### 2.4. Synthesis of the MFe_2_O_4_ (M = Fe, Co, Mn, Zn)

MFe_2_O_4_ (M = Co, Mn, Zn) nanoparticles were prepared via the thermal decomposition of metal acetylacetonates in benzyl ether. Fe(acac)_3_ (2 mmol in case of obtaining Fe_3_O_4_ nanoparticles) or (1.33 mmol in case of obtaining MFe_2_O_4_ nanoparticles), M(acac)_2_ (0.67 mmol), oleic acid (12 mmol), and oleylamine (32 mmol) were suspended in benzyl ether and (40 mL) magnetically stirred under a flow of argon in a 250 mL round-bottomed flask equipped with a magnetic stirrer, thermograph, and heater. The resulting deep red solution was degassed at 110 °C for 30 min. Under argon flow, the temperature of the mixture was increased to 290 °C at a rate of 3 °C/min and was then kept at 290 °C for 2 h. The flask was cooled down to room temperature under an inert atmosphere. Synthesized MFe_2_O_4_ nanoparticles were separated from the reaction media by adding ethanol (4 × 25 mL) as a precipitating agent and separated via centrifugation (6000 rpm, 20 min). The separated nanoparticles were redispersed in toluene (with the required volume in order to obtain the desired concentration), forming a room-temperature stable brown dispersion.

### 2.5. Surface Modification Nanoparticles with DOPAC

In 10 mL of methanol CH_3_OH, 24 mg NaOH was dissolved, followed by the addition of 51 mg of DOPAC. Then, 10 mL of hydrophobic MNPs in toluene (C(Fe) = 0.5 mg/mL) were added to the prepared mixture. For Fe_3_O_4_-Au nanoparticles, an MNPs solution in hexane instead of toluene was used. The mixture was first incubated for 5 h at 50 °C using a water bath under vigorous magnetic stirring and then overnight at room temperature. After cooling the mixture to room temperature, the modified nanoparticles were separated from the supernatant by centrifugation for 20 min at 6000 rpm and redispersed in 10 mL of pure deionized water. Modified nanoparticles were washed three times with pure water using centrifugal filters (Millipore Amicon Ultra-4, MWCO 30 kDa) and separated from any aggregates by passing through 0.45 and 0.22 um syringe filters Millex-HV, successively.

### 2.6. Transmission Electron Microscopy (TEM)

Experiments were conducted using a JEOL JEM-1400 microscope (JEOL, Tokyo, Japan) operated at 120 kV acceleration voltage. Overview images were taken in conventional bright-field transmission mode. Samples were prepared by casting and evaporating a droplet of solution onto a carbon-coated copper grid (300 mesh). The average diameter of MNPs was calculated from TEM images by an analysis of about 500 NPs for each sample using ImageJ software.

### 2.7. Magnetic Properties Analysis

Measurements of static magnetic properties (from −1500 to 1500 kA/m, 300 K) were carried out using a Quantum Design PPPMS-9 (Quantum Design, San Diego, CA, USA with 2 mm amplitude of oscillations, 40 Hz frequency.

### 2.8. Dynamic Light Scattering Studies (DLS)

The hydrodynamic diameter and polydispersity index (PDI) were measured by the dynamic light scattering (DLS) method using a Malvern Zetasizer Nano ZEN3600 (Malvern Instruments Ltd., Malvern, UK). The samples were diluted to a final Fe^3+^ concentration in the range of 0.1–0.3 mg/mL with water and were measured in backscattering mode at 173° at a temperature of 25 °C.

### 2.9. Fourier-Transform Infrared Spectroscopy

The FTIR spectra of samples were registered by means of Nicolet iS20 (Thermo Scientific, Waltham, MA, USA), in KBr, in the range 4000–400 cm^−1^. Infrared spectra were obtained by the KBr pellet method. In this method, the solid sample is finely pulverized with pure, dry KBr, the mixture is pressed in a hydraulic press to form a transparent pellet, and the spectrum of the pellet is measured.

### 2.10. Coating of Fe_3_O_4_-Au MNPs for Drug Delivery

At room temperature, 100 uL 10 mg/mL human serum albumin (HSA), 900 uL PBS (pH = 7.4), and 5 uL FAM-maleimide were mixed for 2 h. Then, 2 mL Fe_3_O_4_-Au MNPs-DOPAC water solution with 0.25 mg [Fe]/mL was mixed with 8 uL N-hydroxysuccinimide (NHS) water solution (1 mg/mL) and 12 uL N-(3-Dimethylaminopropyl)-N′-ethylcarbodiimide hydrochloride (EDC) water solution (1 mg/mL) and incubated at room temperature for 15 min. Then, 100 uL HSA-FAM solution were added, and the resulting mixture was incubated for 12 h at room temperature. Modified nanoparticles were washed five times with pure water using centrifugal filters (Millipore Amicon Ultra-4, MWCO 100 kDa) and separated from any aggregates by passing through 0.45 and 0.22 um syringe filters Millex-HV, successively. The binding between HSA and MNPs-DOPAC was confirmed by Bradford assay: 200 uL Bradford assay solution were mixed with 10 uL of the sample (0.1 mg [Fe]/mL) and incubated for 15 min at room temperature. Then, the optical density was measured using a Multiscan GO plate reader (Thermo Scientific, Waltham, MA, USA), λ = 595 nm, and the HSA concentration was determined via calibration graph.

To improve the stability of the Fe_3_O_4_-Au MNPs-DOPAC-HSA nanoparticles, an additional stabilization was carried out with polyethylene glycol. For this, 2 mL Fe_3_O_4_-Au MNPs-DOPAC-HSA water solution with 0.25 mg [Fe]/mL were mixed with 8 uL NHS water solution (1 mg/mL) and 12 uL EDC water solution (1 mg/mL) and incubated at room temperature for 15 min; then, 54 uL NH_2_-PEG-NH_2_ (PEG, Mn~3000 g/mol) solution in DI water (50 mg/mL) were added, and the resulting mixture was incubated for 12 h at room temperature. The functionalized Fe_3_O_4_-Au MNPs-DOPAC-HSA-PEG were separated from the excess PEG by gel filtration using a PD-10 minicolumn with Sephadex G-25 (eluent–water), followed by filtration using 0.45 um syringe filters Millipore.

### 2.11. Loading of Doxorubicin

Fifty uL of 5 mg/mL doxorubicin hydrochloride (Dox) and 22 uL 10xPBS were added to 1 mL water solution 0.5 mg [Fe]/mL of Fe_3_O_4_-Au@DOPAC@HSA@PEG MNPs and mixed for 4 h. Then, the MNPs-Dox was washed three times with DI water using centrifugal filters (Millipore Amicon Ultra-4, MWCO 30 kDa). The absorption at 480 nm of each of the supernatants was measured and the concentration of Dox was determined. After that, MNPs were separated from any aggregates by passing through 0.45 and 0.22 um syringe filters Millex-HV, successively.

### 2.12. Cytotoxicity Assay

The mouse breast cancer cell line 4T1 (ATCC, Manassas, VA, USA) was cultured in the Roswell Park Memorial Institute 1640 (RPMI 1640) (Gibco, Billings, MT, USA) culture medium supplemented with 10% fetal bovine serum (Gibco), 2 mM L-Glutamine (Gibco), 100 U/mL of penicillin, and 100 ug/mL of streptomycin. Cells were maintained at 37 °C in a humidified incubator MCO-18AC (Sanyo, Moriguchi, Japan) supplied with 5% CO_2_. After attaining 80% confluence, the cells were harvested with TrypLE (Gibco) and subcultured at 1:8. Cell cultures were tested for the absence of mycoplasma.

For cytotoxicity analysis, 4T1 cells were seeded into 96-well plates in 100 uL of growth medium (4 × 10^3^ cells/well). After 24 h of culturing, the growth medium was removed and replaced with substances in PBS at concentrations from 0.4 to 100 ug [Fe]/mL and incubated for 48 h. Then, the growth medium was changed: 20 uL of MTS solution (Promega, Madison, WI, USA) were added to 100 uL of the cell-culture medium into each well. After that, the cells were incubated with the MTS reagent for 4 h and the optical density was measured using a Multiscan GO plate reader (Thermo Scientific, Waltham, MA, USA), λ = 490 nm. All tests were performed in triplicate. All data are displayed as the mean ± SD of three replicates.

### 2.13. Confocal Microscopy

The 4T1 cells were seeded into a Petry dish in 1.5 mL of growth medium (200 × 10^3^ cells/well) and cultured for 24 h. After that, the growth medium was removed and replaced with substances in PBS. The cells were incubated at different times (15, 30, 60, and 120 min) and twice washed using HBSS (with calcium and magnesium ions). Cell imaging was performed using a Nikon Eclipse Ti2 (Nikon, Tokyo, Japan) microscope equipped with a laser scanning system (ThorLabs, Newton, NJ, USA) and Apo 25X/1,1 water immersion objective lenses. Scanning was performed using the ThorImageLS (version 2.4) Software (Thorlabs, Newton, NJ, USA); Fiji software was used to process the images.

## 3. Results

### 3.1. Magnetic Core Synthesis

**Figure 1:**
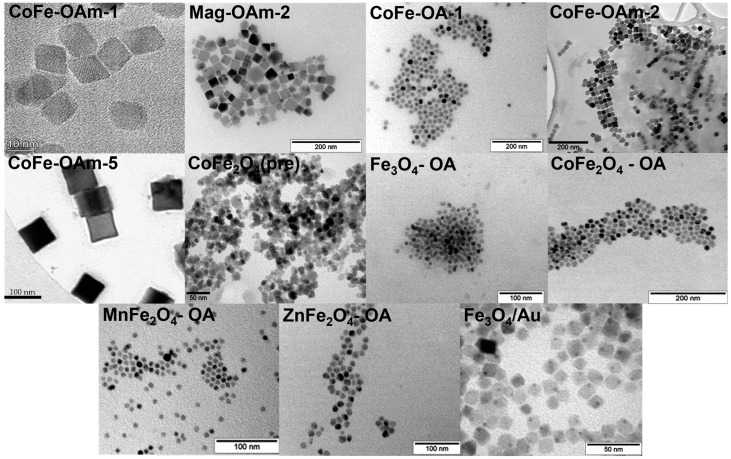
TEM images of the synthesized magnetic cores.

As was previously shown, there is no ideal size and shape of MNPs that will be suitable for every possible application. For example, in the case of application MNPs as T1 contrast agents for an MRI, ultrasmall MNPs with core sizes of less than 5 nm should be used, whereas, for T2 contrast properties, anisotropic MNPs with sizes up to 20 nm are preferential [41,42,43]. On the other hand, for magnetic hyperthermia, anisotropic MNPs (rod- or cube-like morphology) are preferential [44]. In order to demonstrate the versatility of the proposed coating, we decided to synthesize a series of bare MNPs. Magnetite and different ferrite (Co, Mn, and Zn) nanoparticles were obtained by the thermal decomposition method in the presence of oleic acid or its combination with oleylamine. For the dumbbell-shaped Fe_3_O_4_/Au nanoparticles, we used the same chemical approach, and there was the CoFe_2_O_4_ sample, obtained by the coprecipitation technique. The morphology of the nanoparticles was analyzed by TEM, including MNP core-size determination, and the results are presented in Figure 1 and Table 2. Among the received data, one could mention quite a significant range of diameters (from 8 to 100 nm) and various nanoparticle shapes. At the same time, all of them were magnetic, and the corresponding values of saturation magnetization, remanent magnetization, and coercivity were determined (Table S1).

### 3.2. MNPs Stabilization with DOPAC

**Figure 2:**
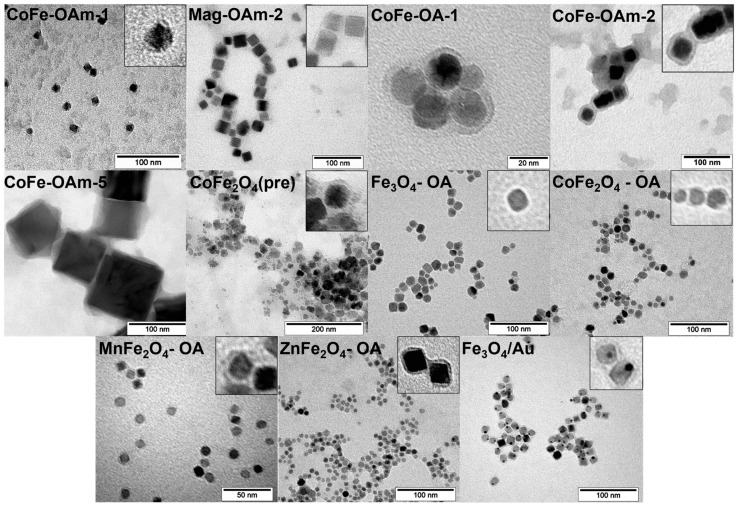
TEM images of the DOPAC-modified magnetic cores.

All types of MNPs obtained at the previous stage were coated by DOPAC in accordance with almost the same procedure. By observing TEM images (Figure 2) of the stabilized nanoparticles, it was possible to notice that small particles were mostly individual, whereas the bigger ones tended to be clustered. There seems to be a certain size limit when dipole–dipole interactions become sufficient for the MNP association. DOPAC-modified nanoparticles were able to form stable colloid solutions in water, which were characterized by the dynamic light scattering (DLS) method (Table 3, Figures S1–S11). The hydrodynamic diameter of nanoparticles increased in comparison with the size determined by the TEM, proving the fact of DOPAC layer formation on the MNP core surface.

It is widely known that nanoparticles for biomedical applications should form stable water colloids. DLS data demonstrate a rather narrow size distribution for all DOPAC-modified MNPs, and hydrodynamic diameters varied from 29 to 307 nm. For NPs with core sizes larger than 20 nm, an increase in hydrodynamic size is observed, indicating the presence of NP aggregates. Except for nanoparticles with core sizes bigger than 20 nm, hydrodynamic diameter values are in the range of less than 200 nm, which is proper for all routes of administration in vivo.

**Figure 3:**
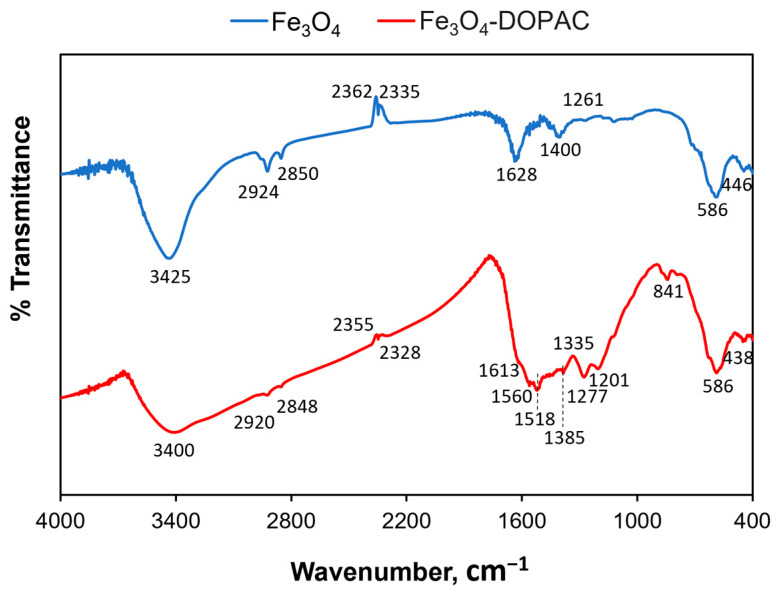
The representative FTIR spectra of the DOPAC-modified and bare Fe_3_O_4_ nanoparticles.

To prove the formation of a DOPAC shell around different magnetic cores, IR spectroscopy was performed. We identified the characteristic peaks that corresponded to initial magnetic cores and chemical bonds specific to DOPAC molecules. Figure 3 represents the IR spectra for magnetite nanoparticles before and after surface modification. Thus, characteristic absorption peaks at 586 and around 440 cm^−1^ refer to the vibrations of Fe-O bonds within the magnetic core, whereas the broad peak around 3400 cm^−1^ is subject to -OH groups on its surface. Moreover, the two peaks at 2924/2920 and 2850/2848 cm^−1^ correspond to the asymmetric/symmetric –CH_2_ stretch and bend of oleic acid residues. Successful DOPAC modification can be confirmed by peak changes in the region of 1200–1600 cm^−1^, where vibrations of carboxyl groups (1560 cm^−1^), aromatic ring (1518 cm^−1^), and aryl C-O (1277 cm^−1^) are represented.

Eventually, the DOPAC layer was successfully formed on the surfaces of both hydrophobic (1–5, 7–10) and hydrophilic (6), together with the dumbbell-like bifunctional Fe_3_O_4_-Au nanoparticles, according to the same synthesis approach.

### 3.3. Doxorubicin Delivery to 4T1 Cells by DOPAC Coated Fe_3_O_4_-Au NP

**Figure 4:**
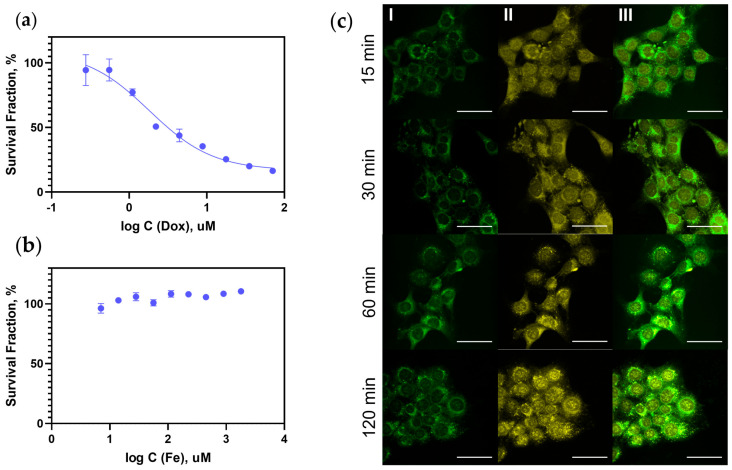
In vitro studies of DOPAC-modified Fe_3_O_4_-Au-DOPAC-HSA MNPs on 4T1 cells: (**a**,**b**)—MTS test results for Dox-loaded and empty MNPs, correspondingly; (**c**) I—Fe_3_O_4_-Au-DOPIHSA-PEG@Dox internalization process in 4T1 cells. I—FAM (fluorescein amidite) fluorescence, corresponding to MNPs, II—Dox fluorescence, III—merge, laser scanning confocal microscopy, scale bar 50 um.

Finally, magnetic nanoparticles stabilized in a water solution by DOPAC layer formation can be conjugated with different types of molecules, such as HSA, which allow for the loading of drugs. We successfully performed doxorubicin (Dox) loading onto the shell of Fe_3_O_4_-Au magnetic cores with a high drug loading capacity (410 ug Dox to 1000 ug Fe) and demonstrated their cytotoxic effect on cancer cells. It is worth mentioning that the proposed procedure was effective for the system of mixed composition: both magnetite and Au nanoparticles were enclosed in a polymeric shell with a stable colloid formation. In order to load Dox, the surface was additionally modified by human serum albumin (HSA) and the resulting nanovehicle was tested in vitro. Thus, cytotoxicity was determined by an MTS test on 4T1 breast adenocarcinoma cells. The IC50 value was 1.8 uM for Dox-loaded magnetic nanoparticles whereas empty nanoparticles did not decrease the viability of living cells in the range of studied concentrations, which corresponded to Fe amount in MNPs with the drug. Based on the data obtained, we can conclude that only Dox is responsible for tumour cell death. Fe_3_O_4_-Au-DOPAC-HSA-PEG@Dox were able to accumulate in 4T1 cells in a time-dependent manner (Figure 4). Dox successfully penetrated the nuclei, where it may disturb the process of DNA synthesis with the following cancer cell death.

## 4. Discussion

The proposed coating with DOPAC is quite universal not only in terms of using different types of magnetic cores but also as a biocompatible layer of core–shell nanoparticles suitable for various biomedical applications. Here, we present some which were demonstrated in our group investigations.

First of all, magnetic nanoparticles have the unique ability to interact with magnetic fields with remarkable consequences. The magnetomechanical approach utilizes this phenomenon in order to manipulate different biological compounds at a single-molecule level. Such a tiny manipulation broadens the horizons for new microarray development, living cell differentiation guidance, enzyme activity regulation, intermolecular interaction measurements, etc. [45]. Thus, DOPAC-modified iron oxide nanoparticles were used for short DNA duplexes detachment from the glass surface in the research carried out by Nikitin et al. [46]. The presence of the DOPAC layer made it possible to cross-link nanoparticles with PEG and single-strand oligonucleotide with the formation of individual probes that are stable in water solutions and able to vibrate under a low-frequency alternating magnetic field. This approach allows for the manipulation of a single DNA molecule or quantifying its interactions within duplexes with another oligonucleotide.

Similarly, cubic magnetite nanoparticles were decorated by DOPAC and then conjugated with PEG and monoclonal antibodies in order to specifically target intracellular biomolecules such as α-tubulin and β-catenin proteins [47]. The coupling with MNPs did not affect the antibodies’ immunochemical functions, which confirmed the flexibility and versatility of the proposed coating. Antibodies-decorated nanoparticles can also be a very useful tool for the precise manipulation of various macromolecules or even cell compartments that can lead to some biochemical activities’ changes. In addition, this technique is able to expand the possibilities of electronic microscopy for various cell components’ visualization, as it works for immunogold labelling [48].

Drug delivery-system development is one of the most important directions of biomedicine. The use of inorganic nanoparticles makes it possible to combine the improvement of drug uptake and the use of the unique physical properties of the particle itself, for example, for hyperthermia, photodynamic therapy, or MRI diagnostics [49]. In particular, in Figure 4, our results show the effective uptake of Fe_3_O_4_-Au nanoparticles and the release of Dox. It should be noted that Fe_3_O_4_-Au nanoparticles are excellent candidates for hyperthermia [50] and theranostics [51,52] and have two unique surfaces in their composition, which can be used to bind two types of molecules with different functions. Early, these nanoparticles were coated by noncovalent interactions between the surface of particles and coating agents such as DSPE–PEG–COOH, Pluronic F127, etc. In this work, we used covalent binding between the Fe_3_O_4_ surface and DOPAC. Also, Fe_3_O_4_-Au-DOPAC nanoparticles were covalently conjugated with HSA and PEG. HSA is an exclusive molecule for drug loading, which elevates the solubility of drug substances under physiological conditions and increases the effectiveness of treatment. For example, there is Abraxane (c), Dox-loaded nanoparticles from HSA [53], etc. In addition, HSA may improve cellular uptake due to the presence of various receptors, such as gp60 (albumin-binding glycoprotein) and SPARC (secreted protein acidic and cysteine rich), which are often located on the cancer cell membrane [54]. Summarizing all of the above, we can assume that Fe_3_O_4_-Au-DOPAC-HSA-FAM-PEG@Dox nanoparticles could be taken up by cells due to receptor-mediated endocytosis. The yellow fluorescence in Figure 4c is localized both in the cell cytoplasm, which matches with the green fluorescence from nanoparticles, and in the cell nucleus. Such a pattern of colocalization is also observed for other types of nanoparticles: HSA-nanoparticles [53], liposomes [55], magnetic nanoparticles [52], PLGA-nanoparticles [56], etc. Fe_3_O_4_-Au-DOPAC-HSA-FAM-PEG nanoparticles show no cytotoxicity effect; however, Dox-loaded nanoparticles lead to a significant decrease of 4T1 cell viability at the same Fe concentrations. The observed effects allow for the conclusion that the presented drug delivery system based on Fe_3_O_4_-Au-DOPAC nanoparticles can be used in further studies for the successful treatment of cancer diseases.

In summary, we report a highly versatile approach to stabilize bare MNPs regardless of their synthetic origin. They may be different in size, shape, synthesis method, or even coupled with other nanoparticles, but finally they will represent stable colloids suitable for following modification steps. Quite often, some specific MNPs are unable to be obtained by any chemical procedure: cubic particles, dimers, or ones that can be produced only as a powder. Consequently, it is extremely difficult to create a number of MNPs grafted by the same polymer. Moreover, it is even more tricky to make them all stable in water suspensions. Fortunately, the proposed DOPAC coating leads to aggregative stable MNPs producing a very thin layer on their surface. On the contrary, DOPAC modification in [57] led to a dramatic increase in nanoparticle size; with the initial, magnetic core of about 6 nm, the hydrodynamic diameter of DOPAC-stabilized MNPs was about 80 nm. Possibly, it could be related to the use of the system of NaOH–methanol in our case instead of ethanol and alkaline water in the presented study. Nevertheless, we believe that the thinner the coating, the less its effect on MNP properties.

On the other hand, the possibility of synthesizing a series of nanoparticles with various parameters but with the same outer shell makes it possible to observe the complex correlations between multiple MNPs’ characteristics with the nanoparticles’ properties (such as relaxivity, cell uptake, biodistribution, etc.) eliminating the coating variable.

Third, an attractive bifunctional chemical nature of DOPAC molecules broadens an opportunity to further modify nanoparticles for various biomedical applications, such as magnetomechanical actuation, drug delivery (chemotherapy, immunotherapy, radiotherapy, etc.), targeting and labelling strategies (coupling with antibodies), MRI, or magnetic hyperthermia. The lateral carboxylic group is highly suitable for bioconjugation under physiological conditions or close to them. In addition, DOPAC is a biocompatible polymer and, thus, fits one of the most important requirements for biomedical nanoparticles.

It is particularly worth noting that DOPAC presents a firmly bonded coating due to chelating iron atoms in the MNPs’ core. Whereas many stabilizing agents form noncovalent bonds with the nanoparticles’ surfaces, these core–shell systems’ stability is questionable. For example, Pluronic, as one of the most common and versatile grafting polymers, is hydrophobically associated with MNPs and may be released because of some change in external conditions or provoke the aggregation process. Additionally, the ligand exchange procedure enables an effective conversion towards hydrophilic MNPs despite the surfactant types used for magnetic core synthesis.

## 5. Conclusions

In our work, we have demonstrated that the proposed technique of MNP stabilization by DOPAC allows for the formation of a stable surface coating for MNPs that were synthesized by coprecipitation or thermal decomposition techniques. During the coating procedure, a DOPAC shell was formed around the magnetic cores with sizes from 8 to 99 nm, which was confirmed both by TEM and DLS measurements. The concept of a universal coating material for a wide range of nanoparticles is extremely relevant for the development of new nanomaterials that are highly suitable for a certain biomedical application route. To find an essential relation between MNP characteristics and their properties, researchers should have the possibility of reducing the number of unpredictable variables. DOPAC coating is a reliable, easy-to-use method of water-dispersible MNP fabrication and facilitates the integration of newly developed magnetic materials into various biomedical applications.

## Figures and Tables

**Table 1:** Composition of the reaction mixture and heating parameters used for the synthesis of CoFe_2_O_4_ and Fe_3_O_4_ MNPs. OA—oleic acid, OAm—oleylamine, 1,2-HDD—1,2-hexadecanediol, DE—dibenzyl ether.

Sample	Fe(acac)_3_, mmol	Co(acac)_2_, mmol	OA, mmol	OAm, mmol	1,2-HDD	DE, mL	T, °C	Time, h	Phase
CoFe-OAm-1	1	0.5	12	2	-	40	280 °C	2	CoFe_2_O_4_
CoFe-OAm-2	2	1	12	2	-	40	280 °C	2	
CoFe-OAm-5	4	2	8	2	-	40	280 °C	4	
CoFe-OA-1	2	1	6	6	-	40	reflux	4	
Mag-OAm-2	0.5	-	8	2	4	10	reflux	4	Fe_3_O_4_

**Table 2:** Morphology and size characteristics of the obtained bare MNPs.

MNPs Core Name	Shape	Size, nm
CoFe-OAm-1	Cubic	14 ± 1
Mag-OAm-2	Cubic	37 ± 6
CoFe-OA-1	Spherical	15 ± 3
CoFe-OAm-2	Cubic	27 ± 4
CoFe-OAm-5	Cubic	99 ± 11
CoFe_2_O_4_ (precipitation)	Spherical	11 ± 2
Fe_3_O_4_-OA	Spherical	11 ± 2
CoFe_2_O_4_-OA	Spherical	16 ± 3
MnFe_2_O_4_-OA	Spherical	8 ± 1
ZnFe_2_O_4_-OA	Spherical	13 ± 2
Fe_3_O_4_/Au	Octahedron/Spherical	14 ± 2/4 ± 1

**Table 3:** Hydrodynamic characteristics of the DOPAC-modified MNPs.

DOPAC Coated MNPs Name	Z-Average, nm	PDI
CoFe-OAm-1@DOPAC	32	0.200
Mag-OAm-2@DOPAC	125	0.300
CoFe-OA-1@DOPAC	307	0.330
CoFe-OAm-2@DOPAC	98	0.340
CoFe-OAm-5@DOPAC	251	0.450
CoFe_2_O_4_(pre)@DOPAC	125	0.220
Fe_3_O_4_@DOPAC	30	0.267
CoFe_2_O_4_@DOPAC	35	0.368
MnFe_2_O_4_@DOPAC	29	0.336
ZnFe_2_O_4_@DOPAC	35	0.353
Fe_3_O_4_-Au@DOPAC	51	0.215

## Data Availability

The data presented in this study are available on request from the corresponding author.

## Supplementary Materials

The following supporting information can be downloaded at: https://www.mdpi.com/article/10.3390/jfb14090461/s1. Table S1: Magnetic properties of the synthesized MNP cores; Figure S1: DLS measurements of CoFe-OAm-1@DOPAC nanoparticles size distribution; Figure S2: DLS measurements of Mag-OAm-2@DOPAC nanoparticles size distribution; Figure S3: DLS measurements of CoFe-OA-1@DOPAC nanoparticles size distribution; Figure S4: DLS measurements of CoFe-OAm-2@DOPAC nanoparticles size distribution; Figure S5: DLS measurements of CoFe-OAm-5@DOPAC nanoparticles size distribution; Figure S6: DLS measurements of CoFe_2_O_4_(pre)@DOPAC nanoparticles size distribution; Figure S7: DLS measurements of Fe_3_O_4_@DOPAC nanoparticles size distribution; Figure S8: DLS measurements of CoFe_2_O_4_@DOPAC nanoparticles size distribution; Figure S9: DLS measurements of MnFe_2_O_4_@DOPAC nanoparticles size distribution; Figure S10: DLS measurements of ZnFe_2_O_4_@DOPAC nanoparticles size distribution; Figure S11: DLS measurements of Fe_3_O_4_-Au@DOPAC nanoparticles size distribution.
