# Supplementary material for: 3,4-Dihydroxiphenylacetic Acid-Based Universal Coating Technique for Magnetic Nanoparticles Stabilization for Biomedical Applications
Source: J Funct Biomater. 2023 Sep 6;14(9):461. doi: 10.3390/jfb14090461 (PMC10531619; doi:10.3390/jfb14090461)
Supplement: Supplementary file 1 [file jfb-14-00461-s001.zip › jfb-2543055-supplementary.pdf]

Supplementary Materials

Table S1. Magnetic properties of the synthesized magnetic cores

**MNP Core**	**Saturation Magnetization J_S, A·m^2/kg**	**Remanent Magnetization J_R, A·m^2/kg**	**Coercivity H_C, kOe**
CoFe-OAm-1	45	14	0.07
Mag-OAm-2	72	14	0.09
CoFe-OA-1	52	26	1.9
CoFe-OAm-2	71	27	1.5
CoFe-OAm-5	81	31	0.8
$CoFe_2O_4$(pre)	62	15	0.37
Fe_3O_4-OA	62	3	0.03
$CoFe_2O_4$ -OA	80	7	0.1
$MnFe_2O_4$-OA	77	7	0.1
$ZnFe_2O_4$-OA	74	7	0.9
Fe_3O_4/Au	64	5	0.07

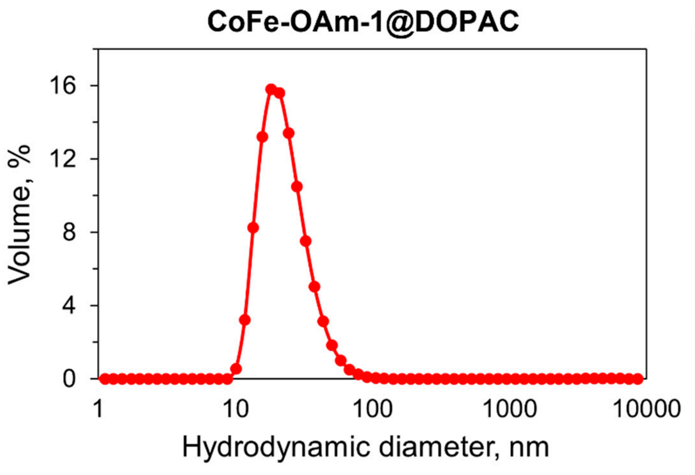

Figure S1. DLS measurements of CoFe-OAm-1@DOPAC nanoparticles size distribution.

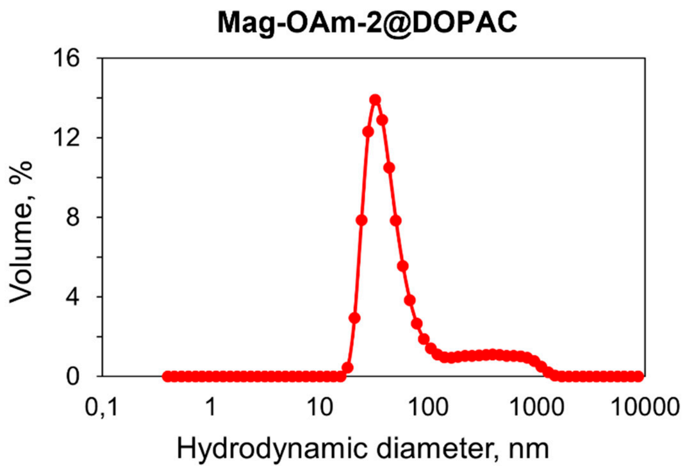

Figure S2. DLS measurements of Mag-OAm-2@DOPAC nanoparticles size distribution.

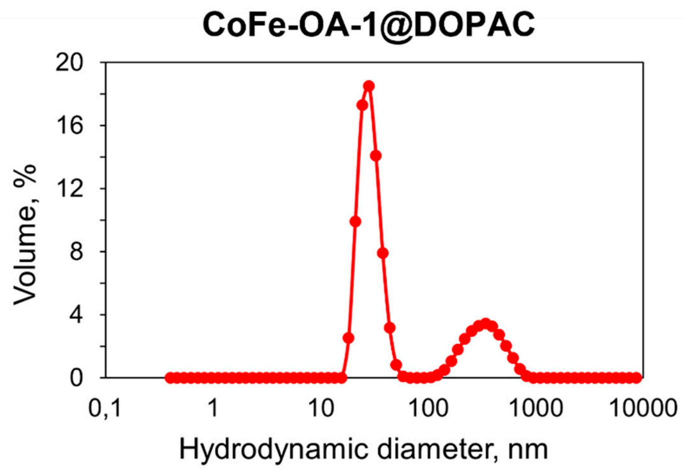

Figure S3. DLS measurements of CoFe-OA-1@DOPAC nanoparticles size distribution.

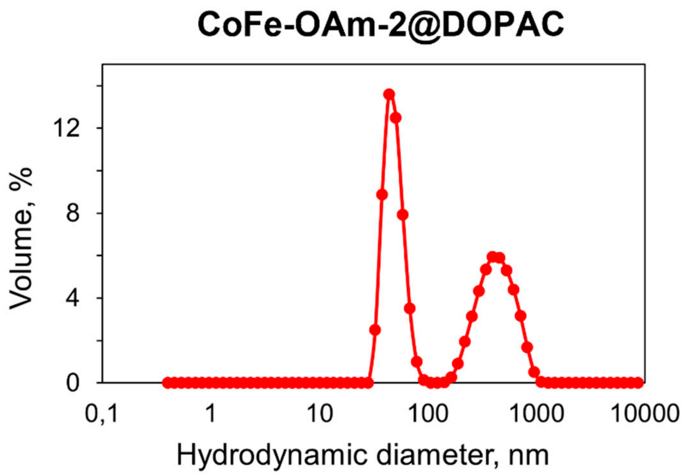

Figure S4. DLS measurements of CoFe-OAm-2@DOPAC nanoparticles size distribution.

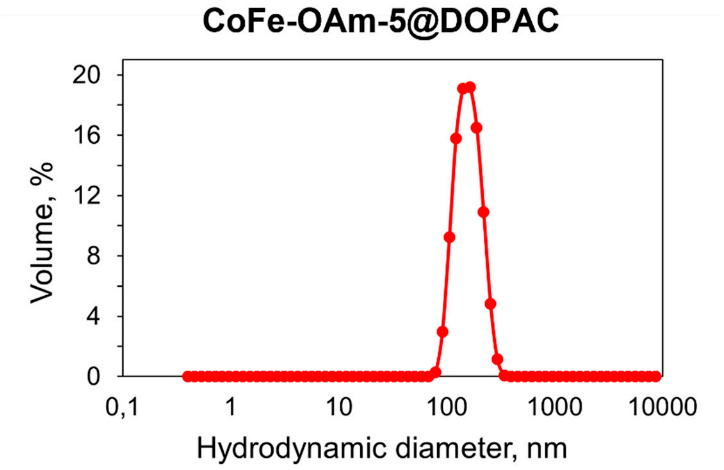

Figure S5. DLS measurements of CoFe-OAm-5@DOPAC nanoparticles size distribution.

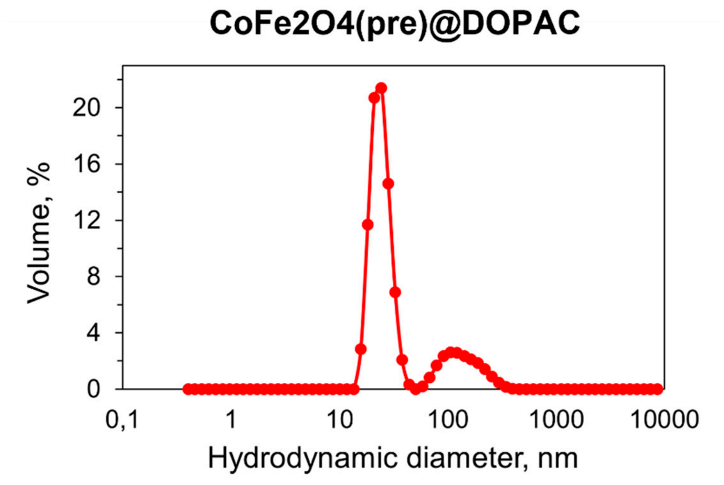

Figure S6. DLS measurements of CoFe2O4(pre)@DOPAC nanoparticles size distribution.

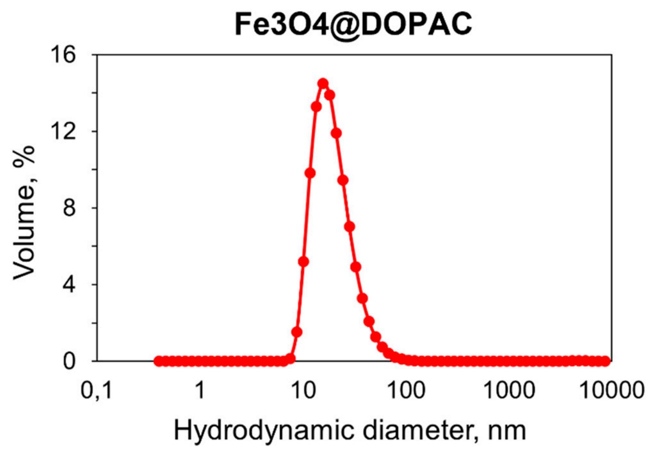

Figure S7. DLS measurements of Fe3O4@DOPAC nanoparticles size distribution.

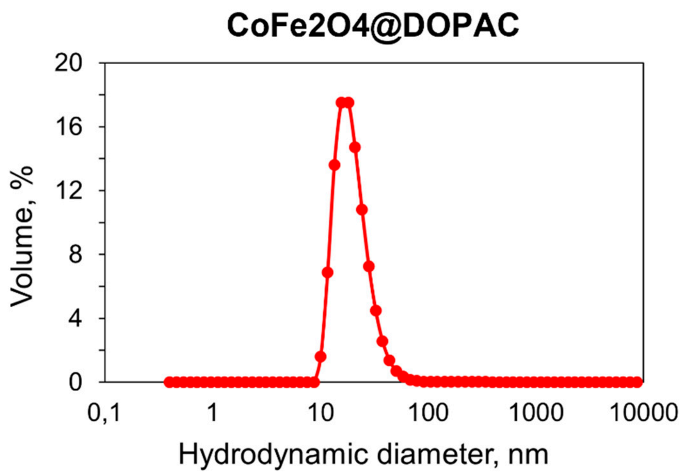

Figure S8. DLS measurements of CoFe2O4@DOPAC nanoparticles size distribution.

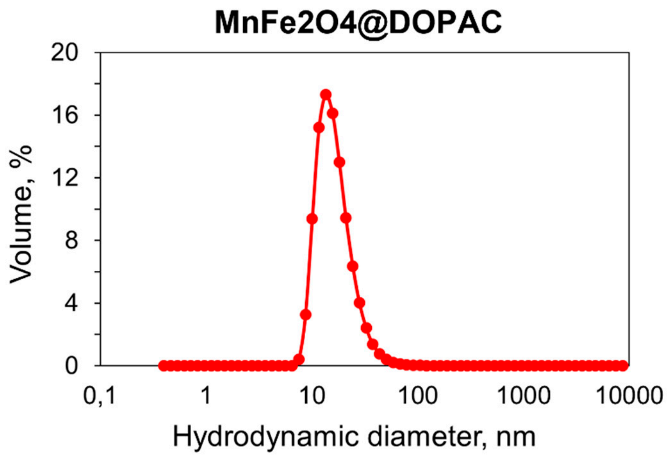

Figure S9. DLS measurements of MnFe2O4@DOPAC nanoparticles size distribution.

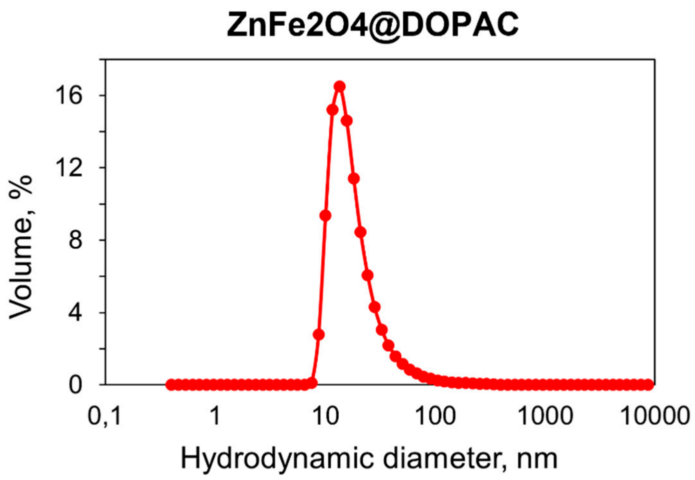

Figure S10. DLS measurements of ZnFe2O4@DOPAC nanoparticles size distribution.

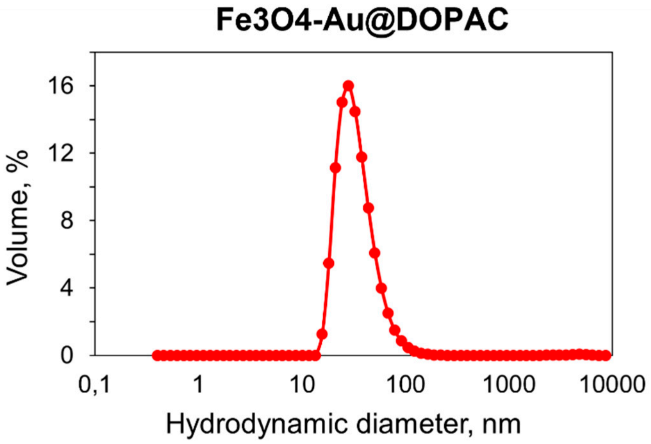

Figure S11. DLS measurements of Fe3O4-Au@DOPAC nanoparticles size distribution.
